# Antioxidative and *in vitro *antiproliferative activity of *Arctium lappa *root extracts

**DOI:** 10.1186/1472-6882-11-25

**Published:** 2011-03-23

**Authors:** Fabricia S Predes, Ana LTG Ruiz, João E Carvalho, Mary A Foglio, Heidi Dolder

**Affiliations:** 1 Department of Anatomy, Cellular Biology, Physiology and Biophysics, Institute of Biology, P.O. Box 6109, University of Campinas, 13083-970, Campinas, SP, Brazil; 2 CPQBA, P.O. Box 6171, University of Campinas, 13083-970, Campinas, SP, Brazil

## Abstract

**Background:**

*Arctium lappa*, known as burdock, is widely used in popular medicine for hypertension, gout, hepatitis and other inflammatory disorders. Pharmacological studies indicated that burdock roots have hepatoprotective, anti-inflammatory, free radical scavenging and antiproliferative activities. The aim of this study was to evaluate total phenolic content, radical scavenging activity by DPPH and *in vitro *antiproliferative activity of different *A. lappa *root extracts.

**Methods:**

Hot and room temperature dichloromethanic, ethanolic and aqueous extracts; hydroethanolic and total aqueous extract of *A. lappa *roots were investigated regarding radical scavenging activity by DPPH, total phenolic content by Folin-Ciocalteau method and antiproliferative *in vitro *activity was evaluated in human cancer cell lines. The hydroethanolic extract analyzed by high-resolution electrospray ionization mass spectroscopy.

**Results:**

Higher radical scavenging activity was found for the hydroethanolic extract. The higher phenolic contents were found for the dichloromethane, obtained both by Soxhlet and maceration extraction and hydroethanolic extracts. The HRESI-MS demonstrated the presence of arctigenin, quercetin, chlorogenic acid and caffeic acid compounds, which were identified by comparison with previous data. The dichloromethane extracts were the only extracts that exhibited activity against cancer cell lines, especially for K562, MCF-7 and 786-0 cell lines.

**Conclusions:**

The hydroethanolic extracts exhibited the strongest free radical scavenging activity, while the highest phenolic content was observed in Soxhlet extraction. Moreover, the dichloromethanic extracts showed selective antiproliferative activity against K562, MCF-7 and 786-0 human cancer cell lines.

## Background

*Arctium lappa *L. (Asteraceae) is a Japanese plant and introduced in Brazil, which is widely used in popular medicine worldwide, as a diuretic and antipyretic tea as well as for hypertension, gout, hepatitis and other inflammatory disorders [1,2]. The root has long been cultivated as a popular vegetable for dietary use and folk medicine [3,4]. *A. lappa *tea has become a promising and important beverage, because of ample therapeutic activity [3]. In the literature, many health benefits have been reported due to different classes of bioactive secondary metabolites. These classes include, among others, flavonoids and lignans, for which *A. lappa *is an important natural source [5]. Pharmacological studies and clinical trials indicated that burdock roots have hepatoprotective [3,6], anti-inflammatory [7] and free radical scavenging activities [7,8] attributed to the presence of caffeoylquinic acid derivatives [9]. Recently, antiproliferative and apoptotic effects of lignans from *A. lappa *were described for leukemic cells [10] as well as antitumor effects of arctigenin on pancreatic cancer cell lines [11]. Consumption of dietary antioxidants from plant materials has been associated with lower incidence of diseases due to reduction of oxidative stress. Thus the aim of this study was to determine the total phenolic content by the Folin-Ciocalteau method, to evaluate the the antiradicalar properties based on their ability to quench the stable radical 2, 2-diphenyl-1-picrylhydrazyl (DPPH) and *in vitro *antiproliferative activity of eight different *A. lappa *root extracts.

## Methods

### Plant material

The roots of *A. lappa *(Asteraceae) were collected at CPQBA, University of Campinas (UNICAMP), experimental field (Paulínia, Brazil) in August 2007. Dr. Glyn Mara Figueira was responsible for identification of the plant species. A voucher specimen was deposited at UNICAMP Herbarium under number 146021.

### Extraction 1

Fresh milled roots (770 g) were extracted successively in a Soxhlet apparatus with dichloromethane, 95% ethanol and water (2:1 solvent/plant ratio), for 6 hours each solvent. The extracts were concentrated under vacuum (Buchi RE 215) until complete elimination of the organic solvent and subsequently freeze-dried for water elimination, providing dichloromethane (DHE), ethanolic (EHE) and aqueous hot extract (AHE).

### Extraction 2

Fresh milled roots (276 g) were successively extracted by dynamic maceration with dichloromethane, 95% ethanol and water (1:5 plant/solvent ratio, 3 times each solvent), at room temperature, in an oscillating agitator (FANEM). The extracts were concentrated under vacuum (Buchi RE 215) until complete elimination of the organic solvent and subsequently freeze-dried for water elimination, providing dichloromethane (DE), ethanolic (EE) and aqueous (AE) extracts.

### Extraction 3

Fresh milled roots (100 g) were extracted three times consecutively in Soxhlet extractor with water (1:5 plant/solvent ratio). The aqueous extract was freeze-dried, providing the total aqueous extract (TAE).

### Extraction 4

Fresh milled roots (594 g) were extracted three times with 70% ethanol (1:5 plant/solvent ratio) under reflux, for 6 hours. The filtrates obtained were combined and concentrated under vacuum. The remaining water was freeze-dried resulting in the hydroethanolic extract (HE).

### High-resolution electrospray ionization mass spectrometry (HRESI-MS) of hydroethanolic extract

HRESI-MS was recorded on a Q-Tof Mass Spectrometer (Micromass - U.K.) using direct infusion of a 10 μL.min-1 MeOH + 0.1% formic acid solution and ionization by electrospray in the negative ion mode. Major operation conditions were as follows: capillary voltage of 3.5 kV, source temperature of 100°C, desolvation temperature of 100°C and cone voltage of 35 V.

### 2, 2-diphenyl-1-picrylhydrazyl (DPPH) radical scavenging activity

Microplate DPPH assay was performed as described by Brand-Williams et al. [12], modified by Brem et al. [13]. Briefly, in a 96-well plate, successive sample dilutions (100 μL/well, 0.25, 2.5, 25 and 250 μg/mL), tested in triplicate, received DPPH solution (40 μM in methanol, 100 μL/well) and absorbance was measured at 550 nm with a microplate reader (VERSA Max, Molecular Devices). Results were determined every 5 min up to 150 min in order to evaluate the kinetic behavior of the reaction. The percentage of remaining DPPH was calculated as follows: % DPPH rem = 100 × ([DPPH] sample/[DPPH] blank). A calibrated Trolox standard curve was also made. The percentage of remaining DPPH against the standard concentration was then plotted in an exponential regression, to obtain the amount of antioxidant necessary to decrease the initial DPPH concentration by 50% (EC_50_). The time needed to reach the steady state for EC_50 _is defined as TEC_50_. The antiradical efficiency [14], was calculated as follows: AE = 1/(EC_50 _× TEC_50_).

### Total phenolic content

The total phenolic content was performed as described by Prior et al. [15], with small modifications in order to use a microplate reader. Briefly, an aliquot (10 μL) of the sample (1 mg/mL) was diluted in distilled water (600 μL). Then, this solution was applied in a 96-well plate (150 μL per well), in triplicate, and received Folin-Ciocalteau solution (12.5 μL), sodium carbonate (37.5 μL, 1 M) and water (50 μL). After incubation at 37°C for 2 h, absorbance was measured at 725 nm with a microplate reader (VERSA Max, Molecular Devices). A calibrated gallic acid standard curve was made and results were expressed as mg equivalents in gallic acid per gram of sample.

### *In vitro *antiproliferative activity assay

Human tumor cell lines UACC-62 (melanoma), MCF-7 (breast), NCI-ADR/RES (ovarian expressing phenotype multiple drug resistance), 786-0 (renal), NCI-H460 (lung, non-small cells), PC-3 (prostate), OVCAR-3 (ovarian), HT-29 (colon), K562 (leukemia) were kindly provided by Frederick Cancer Research & Development Center - National Cancer Institute - Frederick, MA, USA. Stock cultures were grown in 5 mL of RPMI 1640 (GIBCO BRL, Life Technologies) supplemented with 5% fetal bovine serum. Penicilin: streptomycin (1000 μg/mL:1000 UI/mL, 1 mL/L) were added to the experimental cultures. Cells in 96-well plates (100 μL cells/well) were exposed to each extract in DMSO (0.25, 2.5, 25 and 250 μg/mL) at 37°C, 5% of CO_2 _for 48 h. The final concentration of DMSO did not affect the cell viability. Then, a 50% trichloroacetic acid solution was added and after incubation (30 min at 4°C), washing and drying, cell proliferation was determined by spectrophotometric quantification (540 nm) of cellular protein content using sulforhodamine B assay. Using the concentration-response curve for each cell line the TGI (= concentration that produces total growth inhibition or a cytostatic effect) were determined through non-linear regression analysis using the software ORIGIN 7.5 (OriginLab Corporation) and corresponded to the test extract concentration necessary to inhibit proliferation of the cells.

## Results and Discussion

The yields of the different extraction for *A. lappa *are listed in Table 1. The extraction efficiency of the solvents in the successive extractions increased in the order: ethanol > water > dichloromethane. The aqueous and hydroethanolic extraction exhibited the greatest yields.

**Table 1:** Yield of the different solvent extractions of *A. lappa *root

Extract	Yield
Dichloromethane hot extract	0.12%
Ethanolic hot extract	6.39%
Aqueous hot extract	2.87%
Dichloromethanic extract	0.10%
Ethanolic extract	4.45%
Aqueous extract	3.51%
Total aqueous extract	10.56%
Hydroethanolic extract	10.25%

The phenolic compounds are ubiquitous phytochemicals present in plant foods with various biological activities including antioxidant properties. They exert properties such as free radical scavenging and inhibiting the generation of reactive species [16,17]. Phenolic compounds constitute a group of secondary metabolites that are quite widespread in nature with several therapeutical properties [17,18]. Their antioxidant activity is mainly due to their redox properties, which allow them to act as reducing agents, hydrogen donors, free radical scavengers, singlet oxygen quenchers and metal chelators [18].

**Figure 1:**
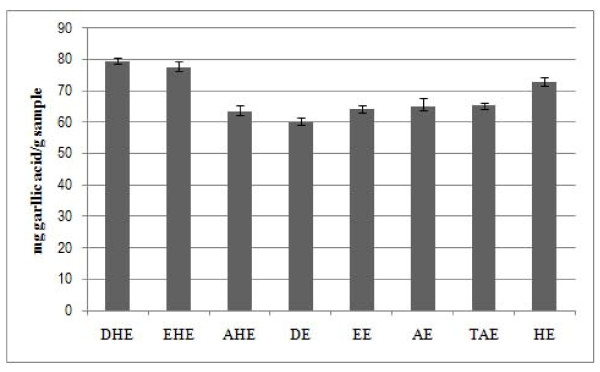
**Total phenolic compounds of *A. lappa *extracts**. DHE: dichloromethane hot extract; EHE: ethanolic hot extract; AHE: aqueous hot extract; DE: dichloromethane extract; EE: ethanolic extract; AE: aqueous extract; TAE: total aqueous extract; HE: hydroethanolic extract. The black bars represent standard deviation.

Total phenolic content of all extracts are shown in Figure 1. The present study showed that the highest phenolic compound concentrations were obtained for Soxhlet extraction with dichloromethane (79.45 mg gallic acid/g extract) and ethanol (77.26 mg gallic acid/g extract) rather than extraction at room temperature. Whereas, the hydroethanolic extract (HE) showed a considerable phenolic content (72.61 mg gallic acid/g extract). A previous study with *A. lappa *roots reported that the extraction with a chloroform and ethanol (1:1) mixture resulted in higher concentration to phenolic compounds (85.15 ± 0.55 mg gallic acid/g dry extract), besides a great quantity of flavonoid (12.57 ± 0.05 mg quercetin/g extract) in the chloroformic extract; moreover, they reported a phenolic content (65.92 ± 0.36 mg gallic acid/g extract) [18] for the ethanol extract which is similar to that described herein. Also, researchers [19] described that *Arctium minus *ssp *minus *leaves aqueous extract exhibited a total phenolic content of 58.93 ± 2.72 mg gallic acid/g of extract, while the ethanolic extract gave 48.29 ± 0.21 mg gallic acid/g of extract.

Many authors have reported a direct relationship between total phenolic content and antioxidant activity in various seeds, fruits and vegetables [20-22]. Antioxidant properties, especially radical scavenging activities, are very important due to the deleterious role of free radicals in foods and in biological systems. The DPPH radical has been widely accepted as a tool for estimating free radical scavenging activities of various compounds and plant extracts [19,21]. Although the present study evaluated the scavenging activity of all extracts, only hydroethanolic extract showed strong antiradicalar activity compared to the commercial standards used, licopene and trolox. Table 2 shows the scavenging effect of extracts and reference substances.

**Table 2:** DPPH radical scavenging of *A. lappa *extract (mean ± SEM)

Sample	**EC**_**50 **_**(μg/ml)**	**TEC**_**50 **_**(min)**	AE
HE	4.79 ± 0.15	5	0.0418 ± 0.001
Lycopene	21.28 ± 0.11	0.1	0.47 ± 0.002
Trolox	1.13 ± 0.1	0.1	8.98 ± 0.84

The hydroethanolic extract of *A. lappa *was then analyzed by high-resolution electrospray ionization mass spectroscopy and the presence of quercetin, arctigenin, chlorogenic acid and caffeic acid was demonstrated. These substances were identified by comparison of their calculated and measured high resolution deprotonated mass (Table 3). Phenolic compounds such as chlorogenic acid, caffeic acid [4], and caffeoylquinic acid derivatives [9] were isolated from *A. lappa *roots. Also, flavonoids such as quercetin and rutin were isolated from leaves [19] and roots [23] of *A. lappa*. Therefore, antioxidant properties of this plant could be attributed to these compounds. Moreover, Erdemoglu et al [19] reported that *A. minus *leaves aqueous extract had antioxidant activity attributed to flavonoids thus corroborating our results for *A. lappa*.

**Table 3:** High-resolution eletrospray ionization mass spectrometry (HRESI-MS) data of Quercetin, Arctigenin, Chlorogenic acid, and Caffeic acid identified in the hydroethanolic extract of *Arctium lappa *root

Compound	Molecular Formule	Calculated [M-H]- mass	Experimental [M-H]- mass	E (ppm)
**Quercetin**	C_15_H_10_O_7_	301.0348	301.0293	18.27
**Arctigenin**	C_21_H_24_0_6_	371.1495	371.1542	12.66
**Chlorogenic acid**	C_16_H_18_O_9_	353.0872	353.0896	6.80
**Caffeic acid**	C_9_H_8_O_4_	179.0344	179.0305	21.78

The antiproliferative properties of the eight extracts of *A. lappa *roots were assessed by using nine human cancer cell lines, and the chemotherapeutic drug, doxorubicin, as a positive control. Among all extracts evaluated, dichloromethane extracts were the only ones with antiproliferative activity. The most active extract (DE) presented a moderate activity for all cell lines with selectivity for K562 (TGI = 3.6 μg/mL) and MCF-7 (TGI = 41.1 μg/mL) (Table 4) while DHE extract displayed the lowest activity with selectivity for K562 (TGI = 17.0 μg/mL) and 786-0 (TGI = 155.7 μg/mL) (Table 4). The difference in the antiproliferative effects between hot and room temperature extractions may have resulted from the different bioactive substances contained in the extracts due to the sensitivity to heat treatment. An antiproliferative activity study, using prostate cancer cells (LNCaP), attributed the inhibitory activity of *A. lappa *seeds hydromethanolic extract to the presence of compounds lappaol A, C and F [24]. A study performed with *A. lappa *showed that dichloromethane seed extract inhibits cancer cell viability under nutrient-deprived conditions, as observed in pancreatic cancer and hepatoma cell lines at 50 μg/ml concentration. The authors also reported the isolation of arctigenin which exhibits cytotoxicity by inducing necrosis in cancer cells [10]. Researchers [11]also reported that hydromethanolic extract of *A. lappa *fruits shows potent antiproliferative activity against B cell hybridoma cells (MH60) attributed to the presence of arctigenin. Ferracane et al [5] recently isolated arctiin from *A. lappa *root, which demonstrated, according to other research groups, a strong cytotoxic effect on human hepatoma cell line (HepG2) [25], human lung cancer (A549), human ovarian cancer (K-OV-3), human skin cancer (SK-MEL-2); human CNS cancer (XF498) and human colon cancer (HCT15) [26].

**Table 4:** Tumor growth inhibition (TGI) (μg/mL) induced by *A. lappa *extracts

	U	M	A	7	4	P	O	H	K
**Doxo**	3,22	0,16	16,79	0,20	0,05	0,34	>25	1,51	**0,03**
**DHE**	>250	>250	>250	155,79	>250	>250	>250	>250	**17,06**
**DE**	**>250**	**41,12**	**>250**	**60,32**	**50,47**	**62,28**	**81,99**	**61,43**	**3,62**

U = UACC-62 (melanoma), M = MCF-7 (breast), A = NCI-ADR/RES (expressing multiple drug resistance phenotype), 7 = 786-0 (renal), 4 = NCI-H460 (lung, non-small cells), P = PC-3 (prostate), O = OVCAR-3 (ovarian), H = HT-29 (colon), K = K562 (leukemia).

*A. lappa *is plant popularly used in the diet as a vegetable and in alternative medicine because it has ample therapeutic action. Moreover, this plant is a component of Flor-Essence^® ^and Essiac^®^, which is two of the most widely used herbal products by cancer patients [27-29]. Several experimental studies have shown evidence of biological activity of *A. lappa *extracts or active compounds including antioxidant, anti-inflammatory, free radical-scavenging, antibacterial and hepatoprotective actions [2]. Thus the current study contributes to the growing literature which demonstrates that *A. lappa *show antioxidant and human tumor cell antiproliferative activities *in vitro*. Although, several studies demonstrated biological properties of *A. lappa in vitro*, further research is needed to elucidate the in vivo activities.

## Conclusions

Our results demonstrated that hydroethanolic extracts exhibited the strongest free radical scavenger activity while the highest phenolic content was observed in Soxhlet extraction with dichloromethane, ethanol and hydroethanolic mixture. Moreover, the dichloromethanic extracts are the most important for this research in that they showed selective antiproliferative activity against K562, MCF-7 and 786-0 human cancer cell lines. On the other hand, the hydroethanolic extract had the greatest yield and shows free radical scavenger activity and high phenolic content, making this extract the best adapted for future "*in vivo*" studies.

## Competing interests

The authors declare that they have no competing interests.

## Authors' contributions

FSP: Was responsible for conception and design, acquisition of data, analysis and interpretation of data and drafted the manuscript. MAF: made substantial contribution to conception and design, interpretation of data and revised it critically for important intellectual content. JEC: made substantial contribution to conception and design of the antiproliferative assay, interpretation of data and revised it critically for important intellectual content. ALTGR: made substantial contribution to conception and design of the antiproliferative, DPPH and total phenolic content assay, interpretation of data and revised it critically for important intellectual content. All authors read and approved the final manuscript.

## Pre-publication history

The pre-publication history for this paper can be accessed here:

http://www.biomedcentral.com/1472-6882/11/25/prepub
